# Caspofungin Inhibits Mixed Biofilms of Candida albicans and Methicillin-Resistant Staphylococcus aureus and Displays Effectiveness in Coinfected Galleria mellonella Larvae

**DOI:** 10.1128/Spectrum.00744-21

**Published:** 2021-10-13

**Authors:** Gaby Scheunemann, Bruna N. Fortes, Nilton Lincopan, Kelly Ishida

**Affiliations:** a Institute of Biomedical Sciences, University of São Paulo, São Paulo, Brazil; Broad Institute

**Keywords:** antibiofilm, polymicrobial infection, echinocandin, micafungin, resistance, MRSA

## Abstract

Candida albicans and Staphylococcus aureus are pathogens commonly isolated from bloodstream infections worldwide. While coinfection by both pathogens is associated with mixed biofilms and more severe clinical manifestations, due to the combined expression of virulence and resistance factors, effective treatments remain a challenge. In this study, we evaluated the activity of echinocandins, especially caspofungin, against mixed biofilms of C. albicans and methicillin-resistant (MRSA) or methicillin-susceptible S. aureus (MSSA) and their effectiveness *in vivo* using the Galleria mellonella coinfection model. Although caspofungin (CAS) and micafungin (MFG) inhibited the mixed biofilm formation, with CAS exhibiting inhibitory activity at lower concentrations, only CAS was active against preformed mixed biofilms. CAS significantly decreased the total biomass of mixed biofilms at concentrations of ≥2 μg/ml, whereas the microbial viability was reduced at high concentrations (32 to 128 μg/ml), leading to fungus and bacterium cell wall disruption and fungal cell enlargement. Notably, CAS (20 or 50 mg/kg of body weight) treatment led to an increased survival and improved outcomes of G. mellonella larvae coinfected with C. albicans and MRSA, since a significant reduction of fungal and bacterial burden in larval tissues was achieved with induction of granuloma formation. Our results reveal that CAS can be a therapeutic option for the treatment of mixed infections caused by C. albicans and S. aureus, supporting additional investigation.

**IMPORTANCE** Infections by microorganisms resistant to antimicrobials is a major challenge that leads to high morbidity and mortality rates and increased time and cost with hospitalization. It was estimated that 27 to 56% of bloodstream infections by C. albicans are polymicrobial, with S. aureus being one of the microorganisms commonly coisolated worldwide. About 80% of infections are associated with biofilms by single or mixed species that can be formed on invasive medical devices, e.g., catheter, and are considered a dissemination source. The increased resistance to antimicrobials in bacterial and fungal cells when they are in biofilms is the most medically relevant behavior that frequently results in therapeutic failure. Although there are several studies evaluating treatments for polymicrobial infections associated or not with biofilms, there is still no consensus on an effective antimicrobial therapy to combat the coinfection by bacteria and fungi.

## INTRODUCTION

Polymicrobial infections caused by bacteria and fungi are recognized with increasing frequency in medical settings (1). In this regard, Candida albicans and Staphylococcus aureus are the most common fungal and bacterial pathogens isolated from bloodstream coinfections worldwide (2, 3). Worryingly, coinfections have clinical implications, limiting therapeutic options, especially when they involve the presence of multidrug-resistant lineages (e.g., methicillin-resistant Staphylococcus aureus, MRSA) and biofilm formation, often associated with higher mortality rates (2, 4–6).

Biofilms are heterogeneous microbial communities attached to biotic or abiotic surfaces, including catheters, and indwelling medical devices that act as infection and dissemination sources. Involved in an extracellular matrix (ECM), biofilms form three-dimensional (3D) structures, where cells have an altered phenotype that differs from their planktonic counterpart, mainly in relation to the reduction of the antimicrobial susceptibility, a key feature that impacts the persistence of infection and contributes to therapeutic failure (7, 8).

While echinocandins (anidulafungin, caspofungin, and micafungin) have been first-line antifungal agents recommended for the treatment of biofilm-associated *Candida* infection (9), effective therapies for established staphylococcal biofilms are not available yet (8, 10). Moreover, there are few studies on treatment strategies against polymicrobial biofilms of C. albicans and S. aureus (10, 11). Therefore, combating single/mixed biofilms is considered a challenge for both researchers and clinicians.

It is worth noting that caspofungin and other echinocandins, combined or not with antibacterial compounds, have shown inhibitory activity against planktonic cells and biofilms of Gram-positive and Gram-negative pathogens (12–17). In fact, previous studies have shown homology of *N*-acetylglucosamine transferase enzymes from S. aureus to β-1,3-glucan synthase enzymes from C. albicans (12). Therefore, in this study, we have evaluated the echinocandin activity, especially caspofungin, against mixed biofilms of C. albicans and methicillin-susceptible S. aureus (MSSA) or MRSA, and the efficacy in the treatment of coinfected Galleria mellonella larvae.

## RESULTS

### Activity of antimicrobials on planktonic cells.

While S. aureus ATCC 29213 and ATCC 6538 were susceptible to all tested antibacterials, S. aureus ATCC 33591 was resistant to all antibacterial agents, except to trimethoprim, confirming a MRSA phenotype (see Table S1 in the supplemental material). Caspofungin (CAS) and micafungin (MFG) inhibited the planktonic cells of C. albicans SC5314 and IAL-40, displaying fungicidal activity, whereas only CAS showed inhibitory and bactericidal activities against S. aureus planktonic cells (Table S2).

### Caspofungin inhibits the mixed biofilm formation and preformed biofilm by Candida albicans and Staphylococcus aureus.

CAS and MFG reduced C. albicans biofilms, both in formation and 24 h preformed. S. aureus biofilms were susceptible to vancomycin (VCM) and both echinocandins. In this regard, CAS inhibited the biofilm formation at lower concentrations than MFG, but only CAS was able to reduce preformed bacterial biofilms (Table S3).

Mixed biofilms formed by C. albicans IAL-40 and S. aureus were more susceptible to echinocandins and VCM (Table 1), most likely due to the lower ability of the IAL-40 strain to form a robust mixed biofilm compared to those formed by the SC5314 strain (Fig. S1). In fact, C. albicans SC5314/S. aureus biofilms were not susceptible to VCM, whereas CAS was slightly more active than MFG against biofilm in formation and the only agent active against 24-h-preformed biofilms (Table 1). Thus, a deeper antibiofilm analysis was performed using CAS, C. albicans SC5314, and S. aureus strains.

**TABLE 1:** BIC of CAS, MFG, and VCM against mixed biofilms (during their formation and 24 h preformed) of C. albicans and S. aureus^*a*^

Biofilm development stage	CAS		MFG		VCM	
	BIC_50_	BIC_90_	BIC_50_	BIC_90_	BIC_50_	BIC_90_
Biofilm formation						
C. albicans SC5314 + S. aureus ATCC 29213	16	128	32	>256	>256	>256
C. albicans SC5314 + S. aureus ATCC 33591	32	64	16	>256	>256	>256
C. albicans SC5314 + S. aureus ATCC 6538	8	32	16	>256	>256	>256
C. albicans IAL-40 *+* S. aureus ATCC 29213	2	64	≤0.125	0.25	1	16
C. albicans IAL-40 *+* S. aureus ATCC 33591	8	128	0.5	16	1	>256
C. albicans IAL-40 *+* S. aureus ATCC 6538	1	32	≤0.125	>256	1	64
24 h preformed biofilm						
C. albicans SC5314 + S. aureus ATCC 29213	128	>256	>256	>256	>256	>256
C. albicans SC5314 + S. aureus ATCC 33591	256	>256	>256	>256	>256	>256
C. albicans SC5314 + S. aureus ATCC 6538	64	>256	>256	>256	>256	>256
C. albicans IAL-40 *+* S. aureus ATCC 29213	64	>256	2	>256	0.5	>256
C. albicans IAL-40 *+* S. aureus ATCC 33591	32	>256	32	>256	8	>256
C. albicans IAL-40 *+* S. aureus ATCC 6538	8	128	16	>256	8	>256

a BIC values are micrograms per milliliter and were assigned as a modal average (*n* = 12).

### Caspofungin reduces the biomass and cell viability of mixed biofilms of Candida albicans and Staphylococcus aureus.

**FIG 1:**
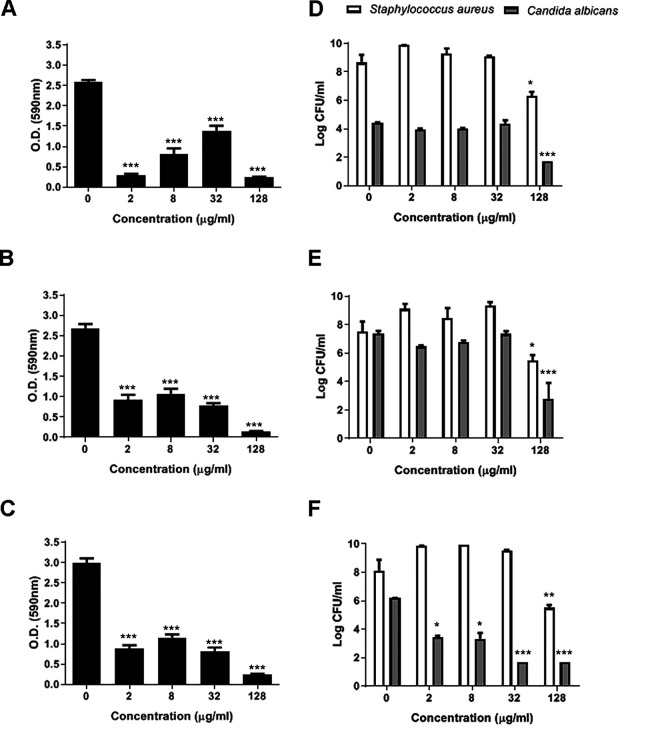
Total biomass (left) and microbial viability (right) of mixed biofilms of Candida albicans and Staphylococcus aureus after caspofungin treatment during biofilm formation. (A and D) C. albicans SC 5314 and S. aureus ATCC 29213. (B and E) C. albicans SC 5314 and S. aureus ATCC 33591. (C and F) C. albicans SC 5314 and S. aureus ATCC 6538. The assays were performed in triplicate at least three times. *, *P* < 0.05, **, *P* < 0.01, and ***, *P* < 0.001 compared with the untreated group (ANOVA one-way followed by Dunnett's test).

CAS treatment resulted in a similar antibiofilm effect against the mixed and single biofilms (Fig. 1, Fig. S2). In this regard, while CAS at ≥2 μg/ml reduced the total biomass of all mixed biofilms (Fig. 1) and single biofilms of C. albicans and S. aureus ATCC 29213 and ATCC 6538 strains, only concentrations of ≥32 μg/ml CAS inhibited the MRSA strain ATCC 33591 (Fig. S2).

The cell viability of C. albicans and S. aureus in both mixed and monoculture biofilms was significantly reduced by 128 μg/ml CAS, except for a single biofilm of S. aureus ATCC 29213 (Fig. 1, Fig. S2). Interestingly, the cell viability of C. albicans in mixed biofilm with S. aureus ATCC 6538 had a greater reduction at ≥2 μg/ml CAS (ca. 50%), whereas only 128 μg/ml CAS was able to reduce the bacterial viability (Fig. 1F).

### Caspofungin induces alterations in the cellular morphologies of mixed biofilms.

**FIG 2:**
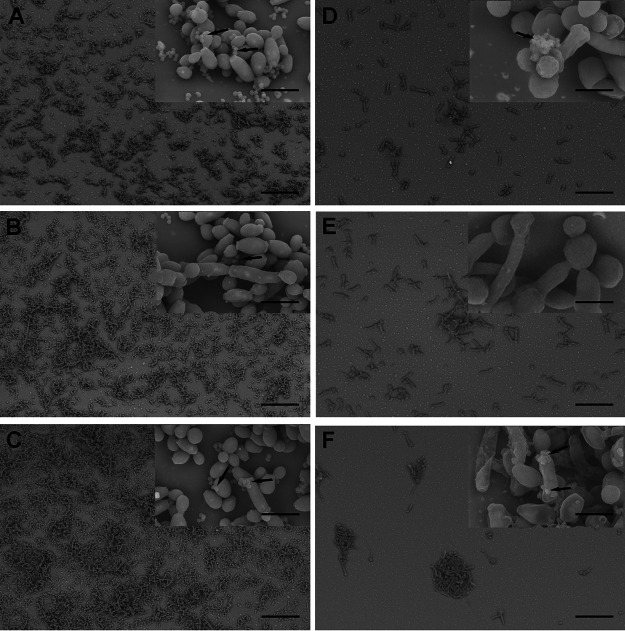
Images of scanning electron microscopy of mixed biofilms of Candida albicans and Staphylococcus aureus left untreated (left) or treated with caspofungin (128 μg/ml) (right). (A and D) C. albicans SC 5314 and S. aureus ATCC 29213. (B and E) C. albicans SC 5314 and S. aureus ATCC 33591. (C and F) C. albicans SC 5314 and S. aureus ATCC 6538. Bars, 50 μm; inset bars, 5 μm.

Cell morphology alterations in mixed biofilms, induced by 128 μg/ml CAS, were monitored, since this concentration significantly reduced viable cells of fungi and bacteria. The untreated mixed biofilms showed microbial cell integrity with bacterial cells adhering on yeast/pseudohyphae of C. albicans as well as on the catheter surface (Fig. 2A to C), whereas CAS reduced adhesion of microbial cells (Fig. 2D to F). Strikingly, MRSA cells were practically absent from the mixed biofilm after CAS treatment (Fig. 2E). Additionally, CAS induced morphological alterations in both pathogens, where bacterial cell wall was disrupted, and fungal cells showed an enlargement and cell wall disruption (Fig. 2D to F).

### Caspofungin effectiveness against coinfected Galleria mellonella larvae.

**FIG 3:**
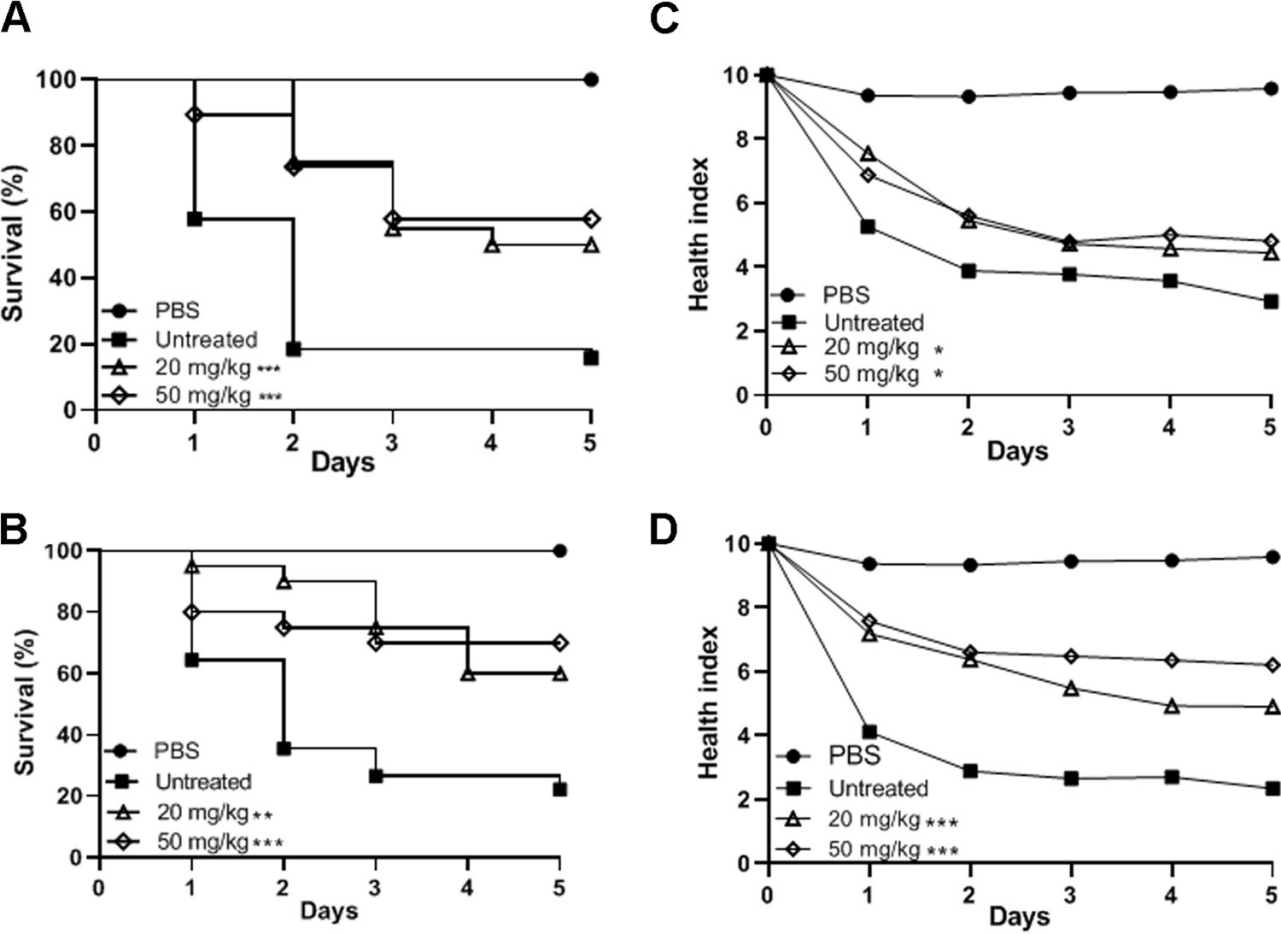
Survival (left) and morbidity (right) curves of Galleria mellonella larvae coinfected with Candida albicans and Staphylococcus aureus and treated with caspofungin. (A and C) C. albicans SC 5314 and S. aureus ATCC 33591 (MRSA). (B and D) C. albicans SC5314 and S. aureus ATCC 6538 (MSSA). *, *P* < 0.05, **, *P* < 0.01, and ***, *P* < 0.001 compared with the respective untreated group (log-rank [Mantel-Cox] test).

**FIG 4:**
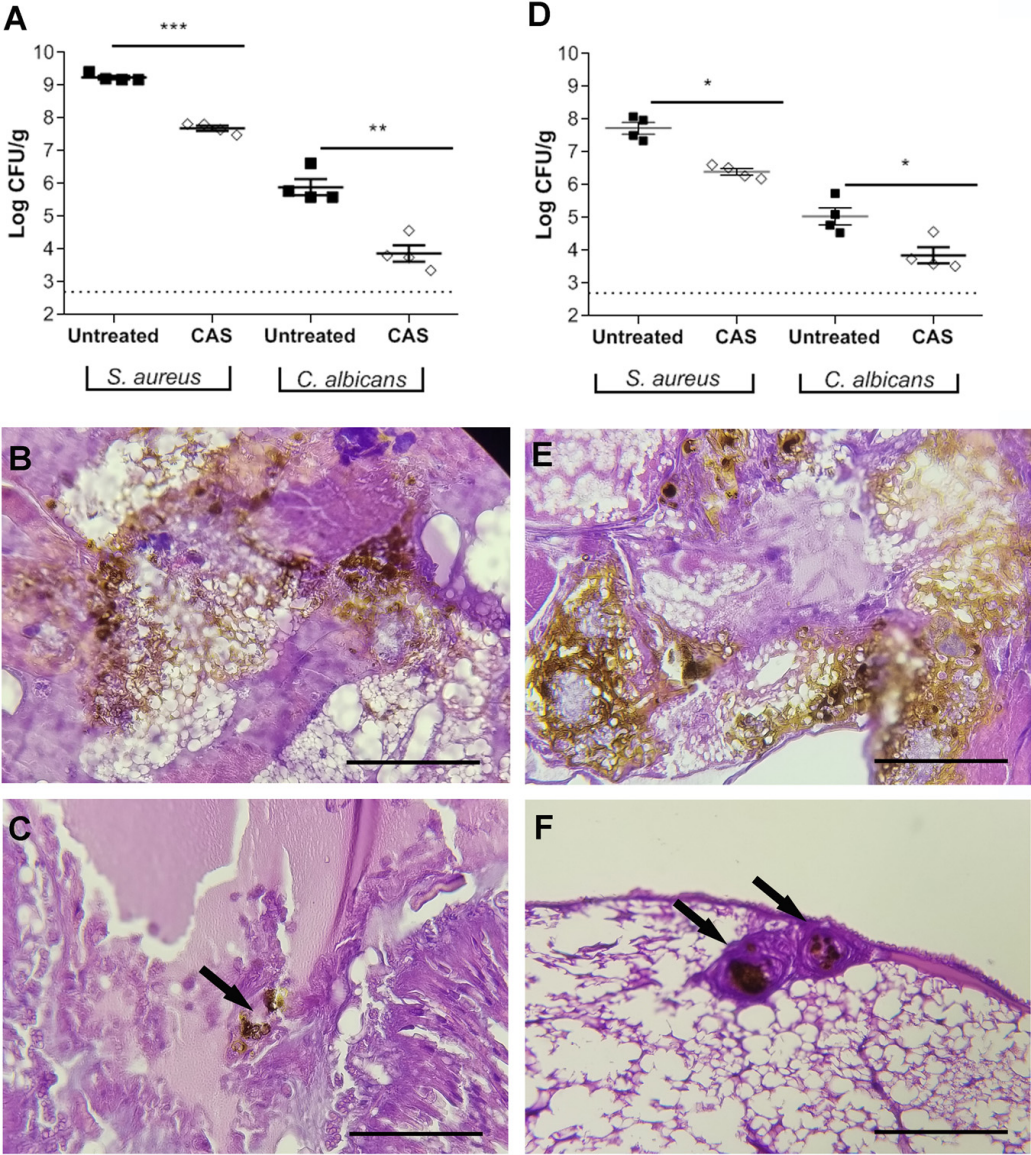
Microbial burden and histopathological analysis of the larval tissues of Galleria mellonella coinfected with Candida albicans and Staphylococcus aureus and treated with caspofungin (CAS) at 50 mg/kg. (A to C) C. albicans SC 5314 and S. aureus ATCC 33591 (MRSA). (D to F) C. albicans SC 5314 and S. aureus ATCC 6538 (MSSA). (A and D) Microbial burden. *, *P* < 0.05, **, *P* < 0.01, and ***, *P* < 0.001 compared with the untreated group (two-way ANOVA). Dotted lines represent the technique detection limit. Histopathology was for samples untreated (B and E) and treated with CAS (C and F); the black arrows indicate the granulomas, and all images were captured at a magnification of ×400. Bars, 25 μm.

Notably, CAS (20 or 50 mg/kg of body weight) treatments increased the health status and survival of larvae coinfected with C. albicans and S. aureus (MRSA or MSSA) compared to the untreated group (*P* < 0.0001) (Fig. 3). In addition, an important and significant reduction in the fungal (∼1 log) and bacterial (1 to 2 logs) burden (Fig. 4A and D) with induction of granuloma formation was observed in the coinfected larvae (arrows in Fig. 4), indicating that CAS contributed to containing the polymicrobial infections in G. mellonella.

## DISCUSSION

Echinocandins are noncompetitive inhibitors of β-1-3-glucan synthase, an enzyme critical to the synthesis of β-1-3-glucan, which is a major component of the fungal cell wall and ECM from C. albicans biofilms. On the other hand, *N*-acetylglucosamine transferase from S. aureus is an important enzyme for synthesis of poly-β-(1,6)-*N*-acetylglucosamine polysaccharide (PNAG; an ECM component) and the polysaccharide intercellular adhesin (PIA) (12). Due to the homology between *N*-acetylglucosamine transferase and β-1,3-glucan synthase, we have investigated the effect of echinocandins, especially CAS, on mixed biofilm formation and preformed biofilm by C. albicans and S. aureus (MSSA and MRSA) and toward coinfection using the Galleria mellonella invertebrate model.

We showed that only CAS showed inhibitory and bactericidal effects on S. aureus planktonic cells, confirming previous studies (12). Inhibitory and bactericidal effects of CAS against planktonic cells of VCM-resistant Enterococcus faecium have also been demonstrated at 32 and 256 μg/ml, respectively (15). Here, both CAS and MFG inhibited the single- and mixed-biofilm formation by C. albicans and S. aureus, but CAS was able to inhibit the biofilm formation at lower concentrations; importantly, only CAS inhibited the preformed mixed biofilms. After CAS treatment, a disruption of cell wall and enlargement of C. albicans were observed as previously described when fungi are treated at high concentrations of CAS (18). Moreover, CAS led to cell wall disruption of S. aureus in the mixed biofilms, corroborating alterations also observed on the E. faecium cell wall in its planktonic form (15).

The interaction of C. albicans and S. aureus is considered synergistic, and the quorum sensing molecules are involved in the cellular communication and provide biofilm formation (2). C. albicans supplies increased bacterial growth and upregulates the virulence factors and antimicrobial resistance (2, 19–21), while S. aureus induces increased C. albicans growth and hypha formation (22, 23). Importantly, mixed C. albicans-Staphylococcus species biofilms display an additional enhanced virulence and tolerance toward antimicrobials compared to their single-species biofilms (2). The interruption of the tridimensional structure formation of biofilms and interkingdom cellular communication then can be considered a relevant strategy for prevention and eradication of mixed biofilms.

The adherence phase is too pivotal for fungus-bacterium interaction and biofilm formation. S. aureus cells predominantly adhere to C. albicans hyphae, resulting in a unique biofilm architecture (4), and the Als3 protein was identified as a hypha-specific receptor that binds bacteria (24); however, other studies showed that C. albicans morphogenesis is not required for their interaction (25). In addition, the interaction can occur between bacteria and yeasts, where cell wall molecules or nonspecific hydrophobic and electrostatic interactions may play a role in interspecies interaction (24, 25). Accordingly, CAS inhibits C. albicans yeast-hypha morphogenesis and interrupts the cell wall biosynthesis of both bacterium and fungus, hampering their cell-cell interaction and, consequently, the 3D structure of biofilms as observed here and in previous studies (12, 26).

The ECM, formed by polysaccharides, proteins, and extracellular DNA, assumes an important role in enhanced tolerance to antimicrobials that physically limit penetration of drugs into the biofilms as well as the persister cell differentiation and upregulation of drug efflux pumps (27). In the mixed biofilms, the presence of C. albicans appeared to protect S. aureus cells from elimination by VCM, an antibiotic normally effective against MRSA, due to the protection of bacterial cells by the ECM produced by C. albicans (28). On the other hand, this protective effect is reduced when the production of β-1,3-glucan was interrupted, facilitating the penetration of VCM into biofilms (28).

CAS directly reduces ECM by inhibition of polysaccharide synthesis in C. albicans (26) and S. aureus (12). In addition, CAS inhibits peptidoglycan synthesis in the Gram-positive bacterium E. faecalis, accumulating muropeptide precursors (15), suggesting that alteration of cell wall composition could also occur in S. aureus cells achieving the cell wall disruption observed here. In this regard, the perturbation of cell wall synthesis in S. aureus induces strong repression of the autolytic system by, e.g., subinhibitory concentrations of β-lactam antibiotics (29), impacting the reduction of eDNA important to the ECM composition. The ECM reduction of biofilms then may directly benefit the antibiofilm activity of antimicrobials such as echinocandins, which act as a facilitator agent for enhanced penetration of drug in the deeper layers of biofilms (12, 14).

Therefore, due to the mechanism of action of echinocandins, they can give an important advantage in the treatment of polymicrobial infections in which *Candida* and Staphylococcus species are involved. Our results showed the effectiveness of CAS in a coinfection model of C. albicans and S. aureus in the G. mellonella larvae, resulting in increased larval survival and reduced fungal and bacterial burden. In murine models, previous studies showed that CAS combined with fluoroquinolones was effective against S. aureus (12), as was anidulafungin combined with tigecycline against S. aureus-C. albicans coinfection (14). It is important to emphasize that the echinocandins are considered safe and well-tolerated antifungal drugs (30).

A limitation of this study was the absence of a murine model to evaluate the effectiveness of treatments. Instead, we used the G. mellonella model. In this regard, new global rules and a modified perception of ethical consciousness have entailed a more rigorous control of utilizations of vertebrates for *in vivo* studies, where numerous alternatives to rodents have been proposed (31). Among these, G. mellonella has played a preponderant role, especially in the microbiological field, as demonstrated by the growing number of recent scientific publications. The reasons for its success must be sought in its peculiar characteristics, such as the innate immune response mechanisms and the ability to grow at a temperature of 37°C (31).

In conclusion, CAS showed a potential effect on the mixed biofilms of C. albicans and S. aureus (MSSA and MRSA strains) in reducing the total biomass as well as the microbial viability. Notably, CAS was able to control the coinfection of C. albicans and S. aureus, increasing the survival and improving the health index of G. mellonella larvae and leading to a relevant reduction of microbial burden. Therefore, our results highlight the potential use of CAS in the treatment of polymicrobial infections by C. albicans and S. aureus; however, further studies should be conducted to refine our findings and improve the therapeutic schemes.

## MATERIALS AND METHODS

### Microorganisms.

Candida albicans (SC 5314 and IAL-40) and Staphylococcus aureus (ATCC 29213, ATCC 33591, and ATCC 6538) were stored in brain heart infusion and tripticaseine soy broths, respectively, with 20% glycerol at −80°C. Yeasts were recovered in Sabouraud dextrose agar and bacteria in tripticaseine soy agar and subcultured in the same medium at least twice at 35°C for 24 h to obtain optimal microbial growth before assays.

### Antimicrobials.

Caspofungin (CAS), micafungin (MFG), and vancomycin (VCM) (all from Sigma-Aldrich Co., MO, USA) were dissolved in dimethyl sulfoxide to obtain 100-times-concentrated stock solutions and stored at −20°C for use in the tests.

### Antimicrobial susceptibility testing for planktonic cells.

The antibacterial profile of S. aureus strains was determined by diffusion disk test, and the broth microdilution assay was performed to determine the MIC of the antimicrobials against planktonic cells of S. aureus and C. albicans (32, 33). The minimum microbicidal concentration was also determined, and it is defined as the lowest concentration that killed 99.9% of microbial cells of the initial inoculum (16).

### Antimicrobial activity on monomicrobial and polymicrobial biofilms.

Here, we tested the antibiofilm effect of antimicrobials in two phases of biofilm development, during biofilm formation and on preformed biofilms, formed by C. albicans (SC5314 and IAL-40) and S. aureus (ATCC 29213, ATCC 33591, and ATCC 6538). The microbial inoculum at 1 × 10^6^ CFU/ml (bacteria and fungi) was standardized in the RPMI 1640 medium buffered with 0.165 M 3-(*N*-morpholino)propane sulfonic acid (here simply called RPMI). A 100-μl aliquot of a single microorganism was dispensed in the 96-well flat-bottomed polystyrene microplate containing 100 μl of RPMI for monomicrobial biofilm formation, and 100 μl of bacteria and 100 μl of fungi were dispensed in the same well for polymicrobial biofilm formation. The microplate then was incubated at 35°C for 1.5 h (adhesion phase) with shaking (150 rpm). Next, the medium was withdrawn, the well washed twice with PBS, and 100 μl of RPMI was added to each well to allow biofilm formation by incubation at 35°C with shaking (150 rpm) for 24 h. Wells with untreated cells (drug-free) and medium alone were used as controls for biofilm formation and medium sterility, respectively. To evaluate antimicrobial activity on biofilm formation, 100 μl of RPMI containing CAS, MFG, or VCM (0.125 to 256 μg/ml) was added to each well after the adhesion phase, and the plates were incubated for 24 h at 35°C, with shaking (150 rpm). To evaluate the effect of antimicrobials on sessile cells of preformed biofilms, the supernatants were removed from each well after 24 h of incubation, and the sessile cells were treated with antimicrobials for 24 h at 35°C, with shaking (150 rpm).

### Violet crystal staining assay.

After antimicrobial treatments, the total biomass of bacterial, fungal, and mixed biofilms was quantified using violet crystal staining (16). The optical density (O.D.) was determined, and the inhibition percentage of antimicrobials was calculated by following the formula 100 − [(treated cells O.D. × 100)/untreated cells O.D.] for determination of the lowest concentrations that inhibit 50% and 90% of biofilm formation (BIC_50_ and BIC_90_, respectively) (16).

### Fungal and bacterial viability.

To evaluate the cell viability in the mono- and polymicrobial biofilms, a CFU counting assay was performed (16). After the adhesion phase, the cells were treated with CAS at 2, 8, 32, or 128 μg/ml in RPMI medium for 24 h at 35°C, with shaking (150 rpm). Next, the sessile cells were washed twice in PBS and removed by scrapping for CFU counts using Sabouraud dextrose agar containing 50 μg/ml chloramphenicol for C. albicans and mannitol salt agar for S. aureus to further calculate of log CFU/ml values (16).

### Scanning electron microscopy.

Mixed biofilms of C. albicans and S. aureus were treated with 128 μg/ml CAS after the adhesion phase on the surface of a catheter section of 5 mm for 24 h at 35°C, with shaking (150 rpm). The biofilms were washed twice with PBS and fixed using 2.5% glutaraldehyde in PBS for 1 h at room temperature. The biofilms then were dehydrated in increasing concentrations of ethanol, dried using hexamethyldisilazane (HMDS; Sigma-Merck), and coated with platinum for observation in a scanning electron microscope (Quanta 650 FEG; FEI, Thermo Scientific, Hillsboro, OR, USA).

### Antimicrobial efficacy of caspofungin against coinfection of C. albicans and S. aureus using the Galleria mellonella model.

C. albicans and S. aureus (MSSA or MRSA) were used for mixed infection in the G. mellonella larvae (ca. 200 mg of body weight) that were obtained in the laboratory at controlled temperature (30°C) using beeswax and pollen as food. For systemic infection, a volume of 10 μl of mixed microbial suspension (5 × 10^5^ CFU for yeast and 1 × 10^7^ CFU for bacteria) in PBS was inoculated in the last larval proleg with a Hamilton syringe. After 30 min of infection, CAS (20 or 50 mg/kg) was administered systemically in another larval proleg for treatments of mixed infections. Infected and untreated larvae (untreated group) and uninfected larvae (PBS group) received only PBS and were included in the assay as control groups for microbial infection and mechanical trauma by injections. A total of 20 larvae were used for each group and incubated at 37°C. The larval survival and health status were monitored every 24 h for up to 5 days after treatments for construction of the survival and morbidity curves, respectively (34). The microbial burden was determined 24 h postinfection (*n *= 4 larvae/group) by CFU counting assay using Sabouraud dextrose agar containing chloramphenicol (50 μg/ml) for *C albicans* and salt mannitol agar for S. aureus to obtain log CFU/g values. The histological analysis was performed using 2 larvae from each group fixed with 4% formaldehyde in PBS and prepared for the histological sections and staining with hematoxylin and eosin (HE) (35).

### Statistical analysis.

Statistical analyses were performed using the software Prism version 8.0 (GraphPad, La Jolla, CA), and *P* values of <0.05 were considered significant.
