# Supplementary material for: Caspofungin Inhibits Mixed Biofilms of Candida albicans and Methicillin-Resistant Staphylococcus aureus and Displays Effectiveness in Coinfected Galleria mellonella Larvae
Source: Microbiol Spectr. 2021 Oct 13;9(2):e00744-21. doi: 10.1128/Spectrum.00744-21 (PMC8515925; doi:10.1128/Spectrum.00744-21)
Supplement: SUPPLEMENTAL FILE 1 — Supplemental material. Download SPECTRUM00744-21_Supp_1_seq2.pdf, PDF file, 0.3 MB [file spectrum00744-21_supp_1_seq2.pdf]

Supplementary Information

Title: Caspofungin inhibits mixed biofilms of *Candida albicans* and methicillin-resistant *Staphylococcus aureus* and displays effectiveness in coinfected *Galleria mellonella* larvae

Authors: Gaby Scheunemann, Bruna N. Fortes, Nilton Lincopan, Kelly Ishida

TABLE S1 Susceptibility of *Staphylococcus aureus* to antibacterial determined by the diffusion disk test.

Antibacterials	*S. aureus* ATCC 29213 (MSSA)	*S. aureus* ATCC 33591 (MRSA)	*S. aureus* ATCC 6538 (MSSA)
Oxacillin	S	**R**	S
Cefoxitin	S	**R**	S
Clindamycin	S	**R**	S
Sulfonamide	S	**R**	S
Trimethoprim	S	S	S
Gentamycin	S	**R**	S
Amikacin	S	**R**	S
Tetracycline	S	**R**	S
Erythromycin	S	**R**	S
Chloramphenicol	S	**R**	S
Ciprofloxacin	S	I	S

S, Susceptible; **I,** Intermediate; **R,** Resistant; **MRSA,** Methicillin-resistant *Staphylococcus aureus*; **MSSA,** Methicillin-susceptible *Staphylococcus aureus*

TABLE S2 Susceptibility of *Candida albicans* and *Staphylococcus aureus* planktonic cells to echinocandins and vancomycin. The values are expressed in µg/ml.

**Strains**	**CAS**			**MFG**			**VCM**		
	IC_{50}	IC_{90}	MMC	IC_{50}	IC_{90}	MMC	IC_{50}	IC_{90}	MMC
C. albicans SC5314	0.06	0.25	2	≤0.03	≤0.03	0.06	n.d.	n.d.	n.d.
C. albicans IAL-40	0.125	0.125	2	0.25	0.25	0.25	n.d.	n.d.	n.d.
S. aureus ATCC 29213	16	32	64	>512	>512	>512	1	1	1
S. aureus ATCC 33591	16	16	64	>512	>512	>512	1	1	2
S. aureus ATCC 6538	8	16	32	>512	>512	>512	0.5	0.5	1

CAS, caspofungin; MFG, micafungin; VCM, vancomycin
IC_{50}, the lowest concentration that inhibiting 50% of microbial growth
IC_{90}, the lowest concentration that inhibiting 90% of microbial growth
MMC, minimum microbicidal concentration
n.d., not determined

TABLE S3 Susceptibility of single biofilms of *Candida albicans* and *Staphylococcus aureus*, during their formation and on 24h-preformed biofilm, to echinocandins and vancomycin. The values are in μg/ml.

Biofilm development stages	CAS		MFG		VCM	
	BIC_{50}	BIC_{90}	BIC_{50}	BIC_{90}	BIC_{50}	BIC_{90}
**Biofilm formation**						
C. albicans SC5314	≤0.125	1	≤0.125	≤0.125	n.d.	n.d.
C. albicans IAL-40	1	2	≤0.125	≤0.125	n.d.	n.d.
S. aureus ATCC 29213	16	64	8	>256	≤0.125	0.5
S. aureus ATCC 33591	8	32	16	>256	≤0.125	1
S. aureus ATCC 6538	2	32	32	>256	0.25	0.5
**24h-preformed biofilm**						
C. albicans SC5314	2	8	1	8	n.d.	n.d.
C. albicans IAL-40	2	4	≤0.125	0.5	n.d.	n.d.
S. aureus ATCC 29213	16	>256	>256	>256	4	8
S. aureus ATCC 33591	32	>256	>256	>256	8	128
S. aureus ATCC 6538	32	64	>256	>256	8	32

CAS, caspofungin; MFG, micafungin; VCM, vancomycin
BIC_{50}, the lowest concentration that inhibiting 50% of biofilm
BIC_{90}, the lowest concentration that inhibiting 90% of biofilm
n.d., not determined

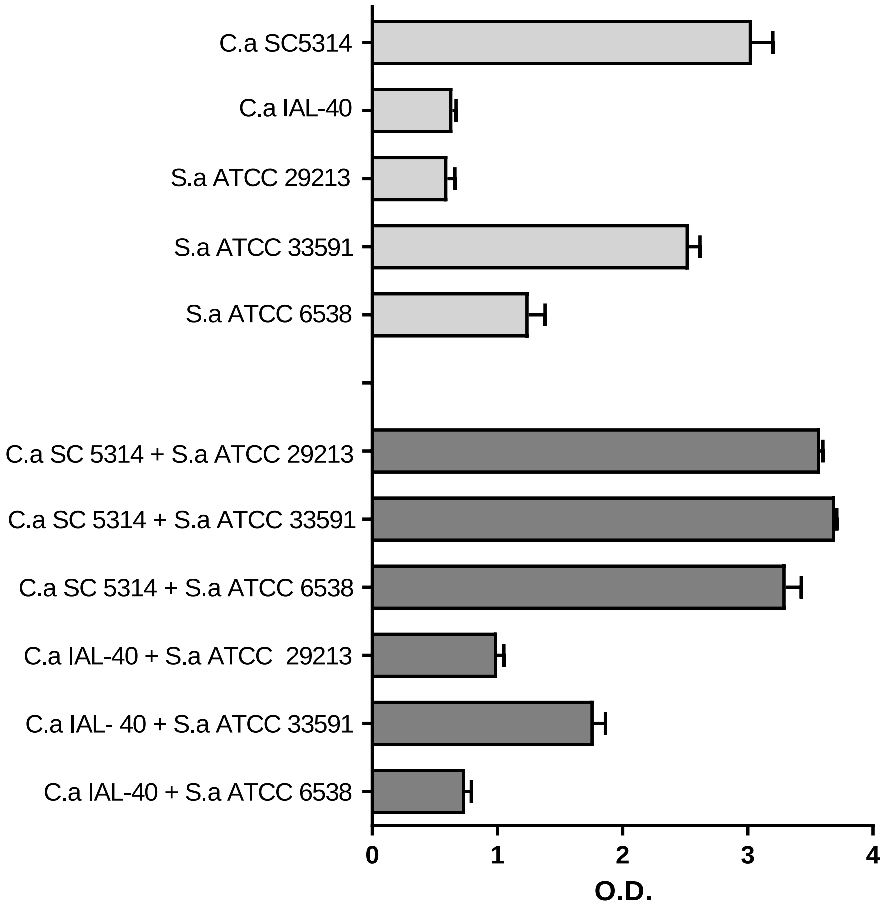

FIG S1 Total biomass of single and mixed biofilms of *Candida albicans* (C.a. SC 5314 and C.a. IAL-40) and *Staphylococcus aureus* (S.a. ATCC 29213, S.a. ATCC 33591 and S.a. ATCC 6538). O.D., optical density.

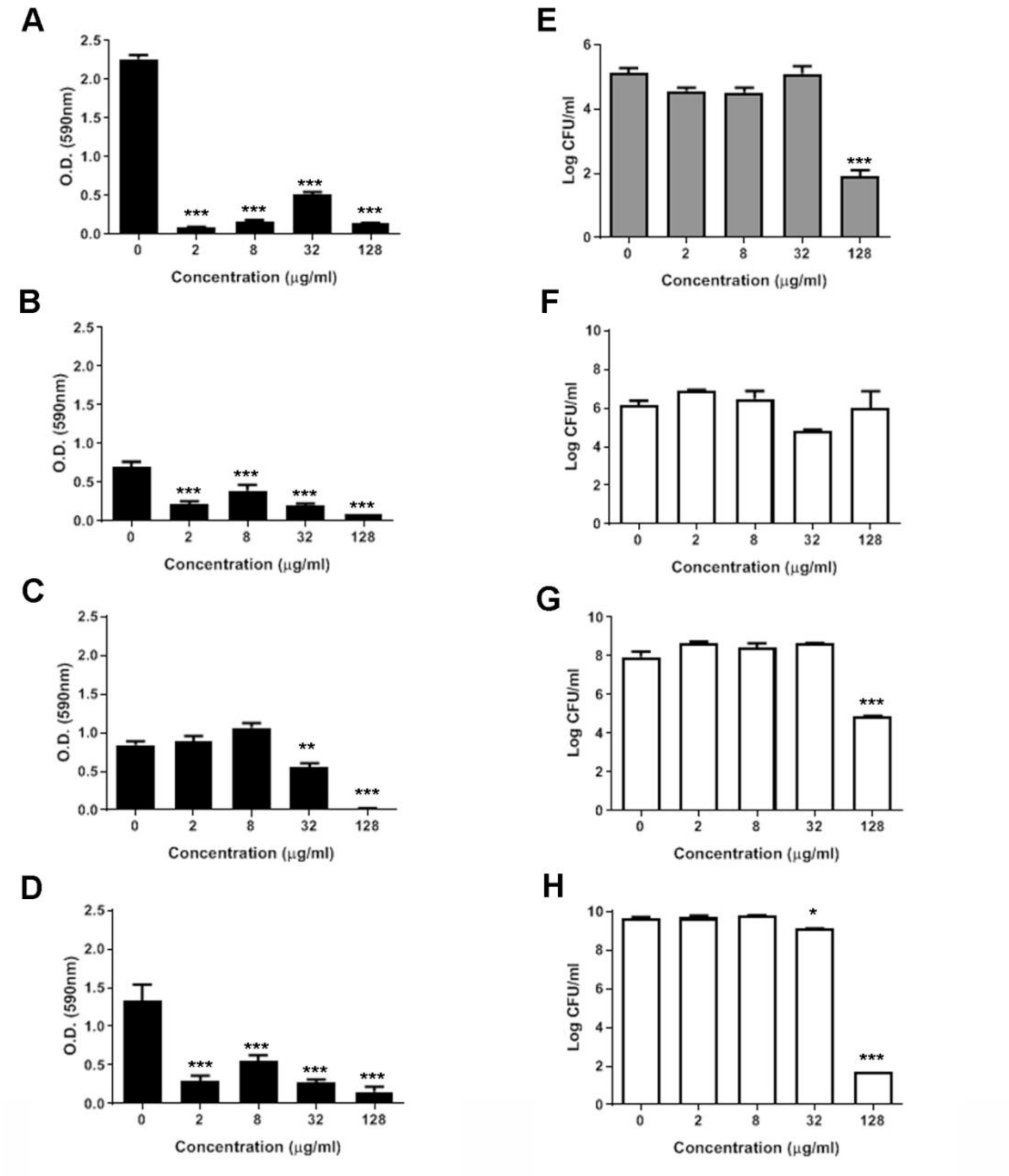

FIG S2 Total biomass (left column) and microbial viability (right column) of single biofilms of *Candida albicans* and *Staphylococcus aureus* after caspofungin treatment during biofilm formation. In A and E, *C. albicans* SC 5314. In B and F, *S. aureus* ATCC 29213. In C and G, *S. aureus* ATCC 33591. In D and G, *S. aureus* ATCC 6538. *p<0.05, **p<0.01, and ***p<0.001 when compared with the untreated group (Anova one-way followed by Dunnett's test).
